# Osteosynthesis using a Brodsky approach after scapular fractures: good clinical results after a mean follow-up of 6.3 years

**DOI:** 10.1186/s13018-025-06657-4

**Published:** 2026-01-18

**Authors:** Malik Jessen, Sebastian Albers, Philipp Zehnder, Michael Zyskowski, Peter Biberthaler, Chlodwig Kirchhoff, Markus Schwarz

**Affiliations:** 1 Department of Trauma Surgery, TUM University Hospital Rechts der Isar, Ismaninger Str. 22, 81675 Munich, Germany; 2 Department of Sports Orthopedics, TUM University Hospital Rechts der Isar, Ismaninger Str. 22, 81675 Munich, Germany

**Keywords:** Brodsky, Posterior approach, Dorsal, Scapula, Osteosynthesis, Clinical-radiological follow-up.

## Abstract

**Background:**

This study evaluated the long-term clinical and radiological outcomes of operatively treated scapular fractures using the tissue-sparing posterior Brodsky approach. We hypothesized that osteosynthesis using this approach would yield favorable functional outcomes.

**Methods:**

This retrospective study included patients with acute scapular fractures treated operatively using the posterior Brodsky approach from January 2015 to December 2019. Radiologic evaluation included fracture classification and fracture union. Functional outcomes were assessed using the Constant-Murley Score (CMS), Disabilities of the Arm, Shoulder, and Hand (DASH) questionnaire, the Shoulder Pain and Disability Index (SPADI), Visual Analogue Scale (VAS), and range of motion. Postoperative complications were documented.

**Results:**

A total of 16 patients with a mean follow-up of 6.3 years were included. The cohort included both extra-articular and intra-articular fracture patterns, with associated injuries such as coracoid fractures. All fractures achieved radiographic union. The mean Constant–Murley Score was 75 ± 14 points, the SPADI score was 85 ± 15, the DASH score was 15 ± 15, and the mean VAS score was 2 ± 1. Shoulder motion was largely preserved, with a mean external rotation of 70° ± 12°. Postoperative complications were observed in one patient due to incorrect screw placement during coracoid fracture fixation. Five patients required a staged surgical procedure due to associated coracoid fractures.

**Conclusions:**

Operative treatment of scapular fractures using a tissue-sparing posterior approach was associated with favorable long-term functional outcomes, preserved shoulder motion, low pain levels, and reliable fracture union, with a low complication rate, even in cases requiring staged procedures due to associated injuries.

## Introduction

 Fractures of the scapula typically result from a direct, blunt, and forceful impact and account for three to five % of all shoulder girdle fractures [1]. They may also be associated with intra-articular trauma involvement [2]. Scapular body fractures can be managed conservatively, involving a brief immobilization period followed by motion and strengthening exercises [3]. The indication for surgical intervention is not yet clearly defined in the current literature [4]. In addition to displaced process fractures, surgery is generally indicated for intraarticular fractures with a step-off of > 2–5 mm and glenoid neck fractures with > 20–30° angulation [5, 6], media/lateral displacement of > 20 mm, medial/lateral displacement of > 15 mm with angulation of > 30°, double disruption of the superior shoulder suspensory complex (SSSC) both displaced > 10 mm and a glenopolar angle (GPA) of < 22° [4, 7, 8]. For displaced glenoid rim fractures following anterior shoulder dislocation, either an arthroscopic or deltopectoral approach may be used; however, for displaced glenoid and neck fractures, a posterior approach is typically preferred [3, 6].

Several dorsal approaches to the scapula have been described, including the classic and modified Judet approaches, which offer excellent exposure but may require detachment of the posterior deltoid, increasing the risk of soft-tissue morbidity [5, 9–13]. In contrast, the Brodsky approach preserves the deltoid and external rotators and has shown favorable short-term outcomes in extra-articular fractures [14, 15]. Nevertheless, concerns persist regarding its visualization in complex fracture patterns [1, 16], and evidence on long-term outcomes, particularly for fractures with intra-articular involvement, is limited.

The present study evaluates the long-term clinical and radiological outcomes of scapular fracture fixation using the Brodsky approach, including cases with complex fracture patterns. We hypothesized that this tissue-sparing approach would yield good functional results, particularly in terms of range of motion (ROM) and external rotation.

## Methods

A retrospective single-center review was performed of all patients with scapular fractures treated operatively at our Level I trauma center between January 2015 and December 2019. Institutional Review Board approval was obtained before the initiation of the study (Ethics Committee of the Technical University of Munich, Germany, 2023-6-S-NP), and all patients provided informed consent. Reporting followed the STROBE guidelines [17].

### Patient selection

The inclusion criteria were an acute scapular fracture (less than 3 weeks), operative treatment via the Brodsky approach, age 18–80 years, clinical follow-up of at least 24 months, and radiographic follow-up of at least 12 months. Exclusion criteria were alternative dorsal approaches, prior ipsilateral shoulder surgery, pathological fractures, and vulnerable populations (minors, pregnant, incarcerated, or declining participation).

The indication for operative treatment was based on commonly accepted criteria reported in the literature [4, 6, 18, 19]. Surgery was reserved for displaced or unstable scapular fractures, including relevant body fractures with medial–lateral displacement of the lateral border ≥ 20 mm and/or angular displacement ≥ 45°, involvement of the glenoid or scapular neck with displacement ≥ 1 cm and/or angular displacement ≥ 40°, intra-articular step-off or gap ≥ 4 mm, instability of the superior shoulder suspensory complex (SSSC) with displacement ≥ 10 mm, pathological glenopolar angle (GPA) ≤ 22°, or open fractures.

### Surgical technique

**Fig. 1:**
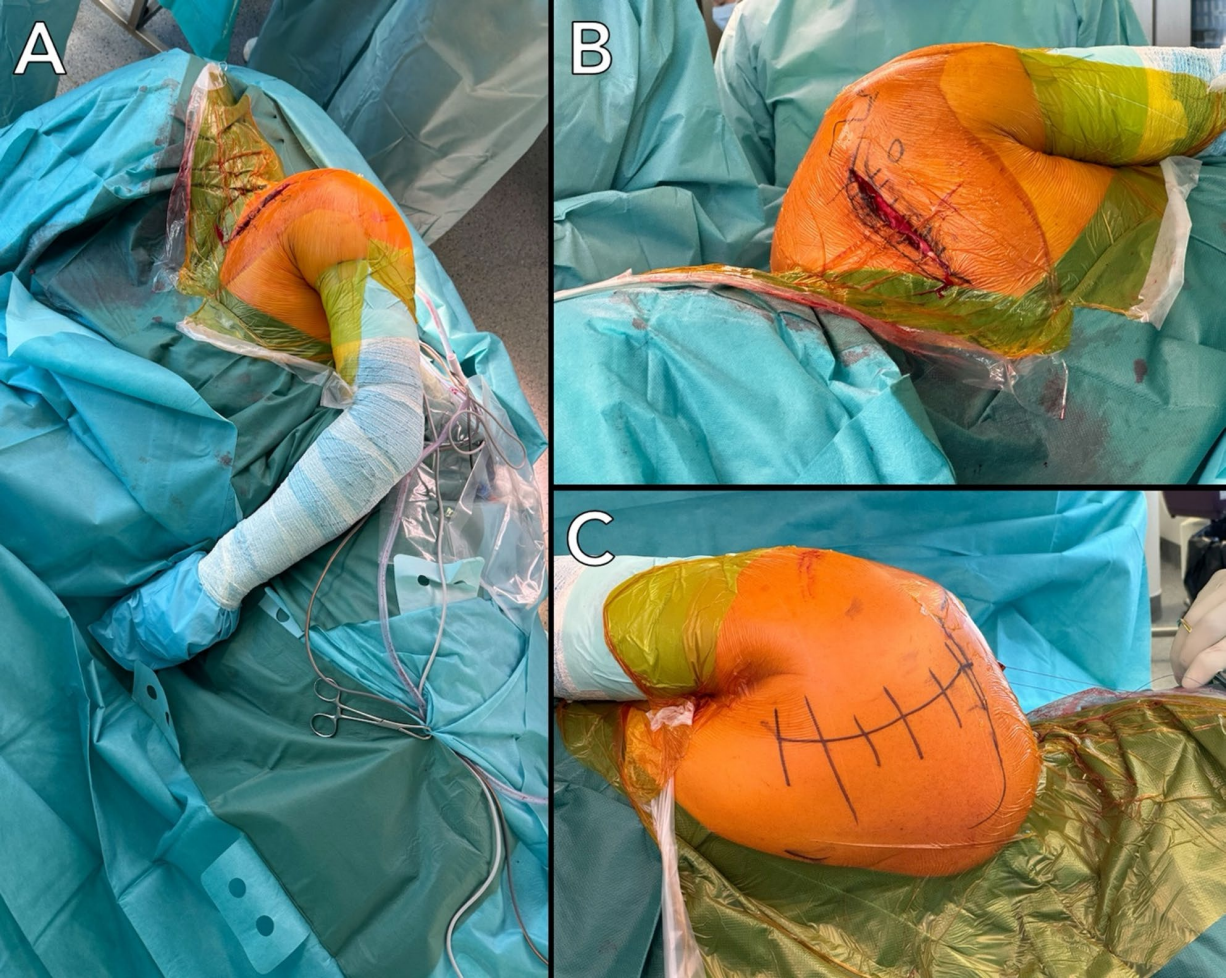
Patient positioning in the lateral decubitus position (**A**). An associated clavicle fracture can be addressed through an anterior approach (**B**) without intraoperative repositioning. The dorsal aspect of the scapula allows access for the posterior Brodsky approach (**C**)

Surgery was performed by one senior surgeon (CK). All patients were positioned either in the lateral decubitus or in a prone position (Fig. 1).

**Fig. 2:**
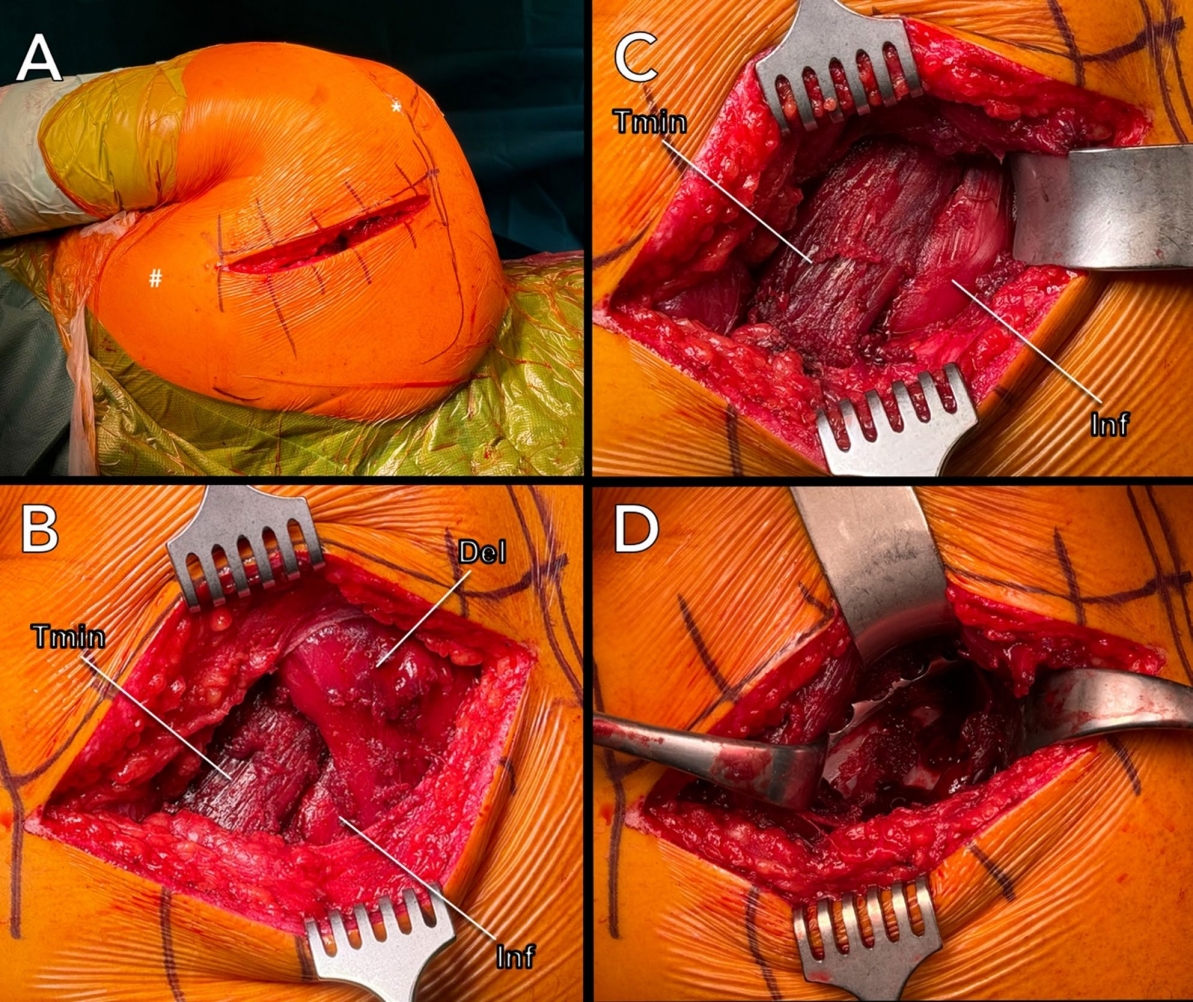
**A–D**: Illustration of the posterior scapular approach according to Brodsky. **A** Skin incision; (*) Acromion, (#) inferior angle of the scapula. **B** Subfascial exposure of the posterior deltoid (Del); (Tmin), teres minor muscle, (Inf) infraspinatus muscle. **C** Interval between the (Inf) and (Tmin). **D** Visualization of the scapular fracture

The patient was placed in a manner that allowed complete access to the entire scapula dorsally and the clavicle ventrally (Fig. 1A). Throughout the entire surgical procedure, the patient’s arm remained in a neutral position. An incision was made starting 3–5 cm medial to the lateral acromion edge and 2 cm caudal to the spine of the scapula (Fig. 2A).

The skin incision was approximately 10–15 cm long and directed caudally. Following epifascial dissection, the posterior border of the deltoid is identified (Fig. 2B). The posterior portion of the deltoid muscle is elevated with no need for detachment (Fig. 2C). The interval between the infraspinatus and teres minor is developed typically following the infraspinatus fascia. The infraspinatus is retracted medially, and the teres minor is retracted laterally (Fig. 2D). Thus, exposing the margo lateralis, the scapular neck, and, if required, the posterior aspects of the glenoid. Detachment of the posterior rotator cuff or the posterior joint capsule does not have to be performed unless an intra-articular fracture is present. In case of a severe displaced intra-articular fracture, Capsulotomy through the dorsal approach (between the supraspinatus and infraspinatus muscles) has to be performed along the posterior border of the glenoid to expose the glenohumeral joint. In cases with associated coracoid fractures, a staged surgical approach was employed. Fixation of the scapular fracture via the Brodsky approach was performed in the prone position. In contrast, coracoid fixation required a separate anterior procedure with the patient positioned in the beach-chair position to allow adequate anterior exposure. When an associated intra-articular glenoid fracture was present, the anterior approach was used to address the intra-articular component during the same stage, provided that sufficient exposure could be achieved.

By contrast, the presence of an associated clavicle fracture was not considered an indication for staged surgery. In these cases, clavicle fixation was performed through an anterior approach during the same operative session while the patient remained in the lateral decubitus position, without the need for intraoperative repositioning (Fig. 1).

**Fig. 3:**
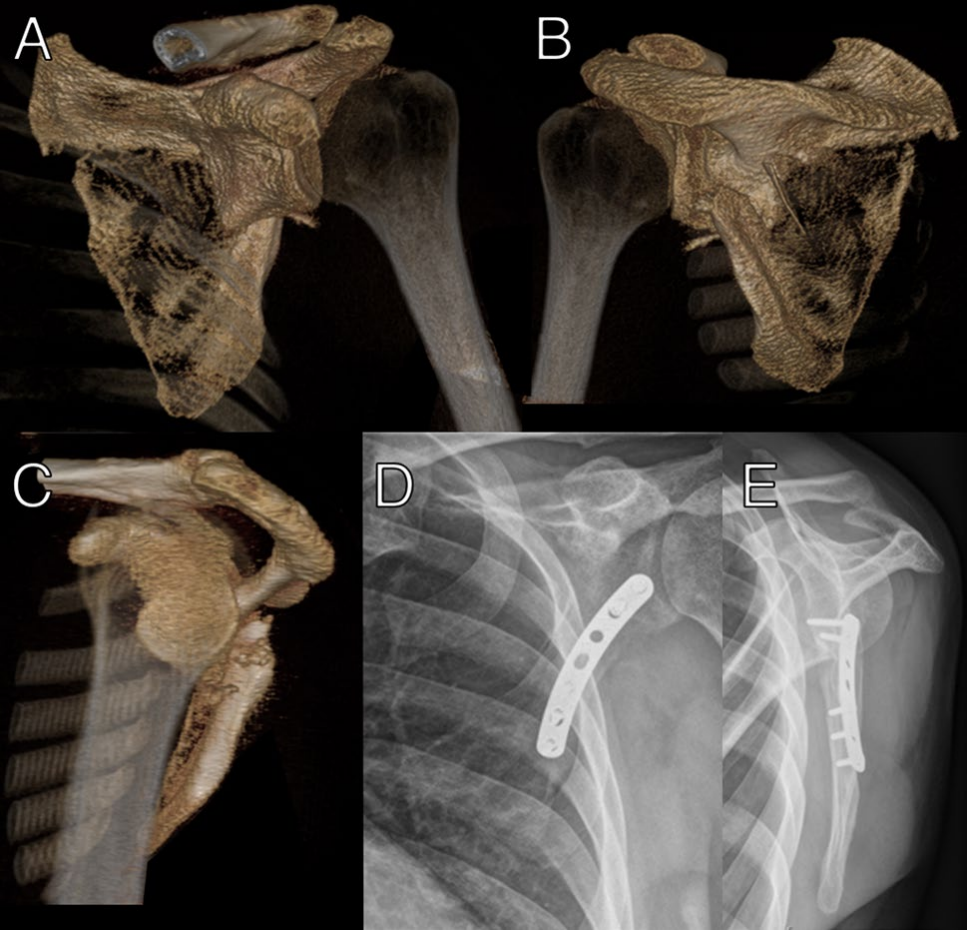
**A–E**: Preoperative 3D reconstruction showing a scapular body fracture with coracoglenoid block formation (**A**–**C**). Postoperative anteroposterior (**D**) and Y-view (**E**) radiographs following ORIF

**Fig. 4:**
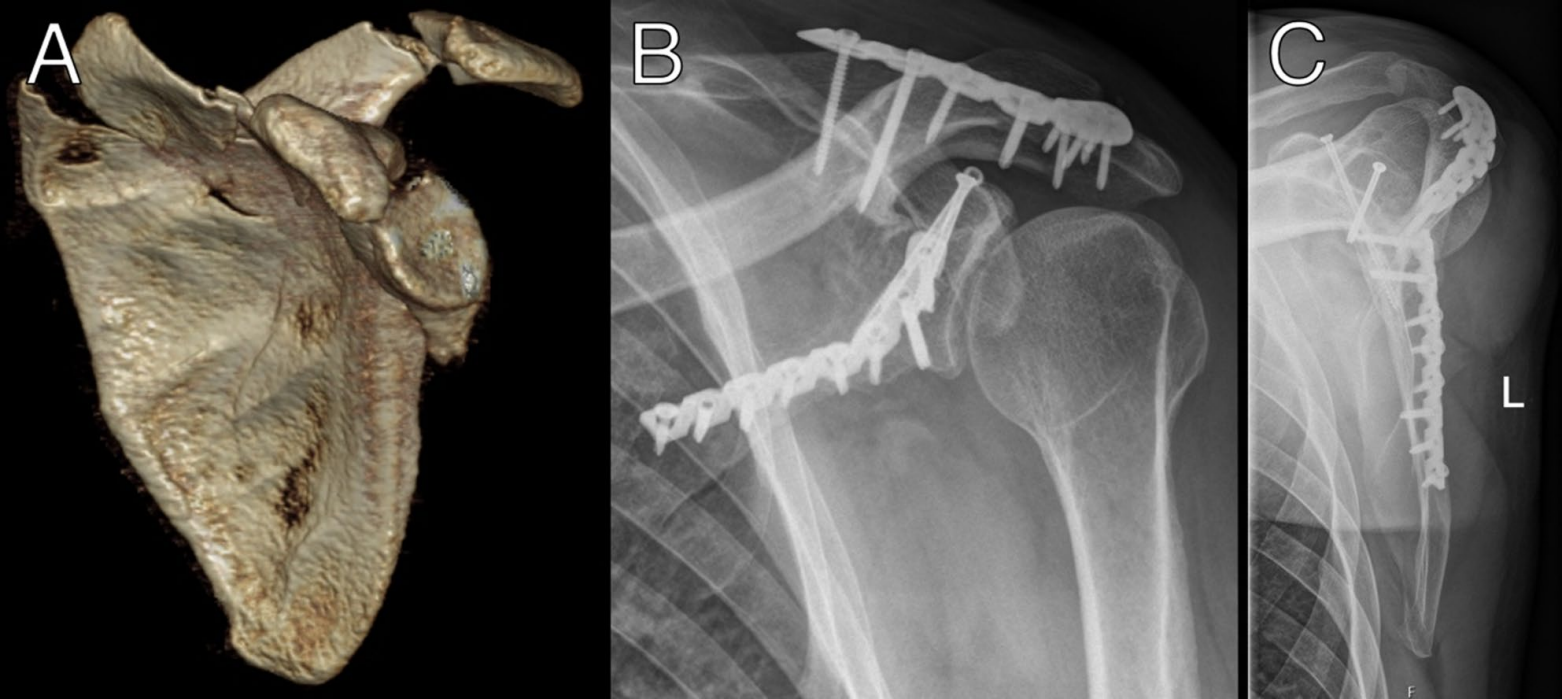
**A–C**: Preoperative 3D reconstruction of a scapular body, glenoid, and spine fracture (**A**). Postoperative anteroposterior (**B**) and Y-view (**C**) radiographs after surgical treatment

Instability was not assessed using a standardized stress test. Instead, surgical decision-making was guided by objective preoperative radiological findings, including fracture pattern, degree of displacement, involvement of the superior shoulder suspensory complex, and associated injuries such as coracoid fractures. Intraoperatively, instability was assessed qualitatively based on visual inspection and manual evaluation of fracture mobility and alignment after exposure. Formal instability testing was not routinely performed, as reproducible testing in prone or lateral positioning is limited. No additional bone grafting was used. Internal fixation of the scapular fracture is further performed using a clavicle plate (Arthrex, Naples, U.S.A.; Fig. 3) in 13 cases, with an LCP plate (DePuy Synthes, Raynham, U.S.A.) in 2 cases, and a combination of both plates (Fig. 4B, C) in 2 cases.

Indirect reduction techniques, including the use of a temporary plate, a free buttress screw, and a laminar spreader, were employed to counteract substantial shortening forces and restore anatomical length. Fascia and skin were closed in a standard fashion.

### Postoperative rehabilitation

Rehabilitation consisted of passive abduction and flexion to 90° and active-assisted external rotation to neutral for the first three postoperative weeks. Between weeks 4 and 6, passive and active-assisted ROM up to 90° was permitted. From week 7 onward, unrestricted active-assisted ROM was allowed, with full active mobility after week 9. Clinical and radiographic follow-up occurred at 6, 12, and 52 weeks.

### Clinical evaluation

Functional outcomes were assessed using the Munich Shoulder Questionnaire (MSQ) [20], the Constant-Murley Score (CMS), the DASH, the SPADI, and the VAS for pain. Muscle strength was assessed as part of the CMS and reflects abduction strength according to the standardized CMS protocol. Active ROM (flexion, abduction, external rotation at the side) was measured with a goniometer; internal rotation was documented as the highest reachable vertebral level.

### Radiographic evaluation

Preoperative imaging included standardized shoulder radiographs (anteroposterior and lateral scapular Y-views) and CT scans with 3D reconstruction. Postoperative evaluation was based on available radiographs and CT images stored in the institutional database and was performed by one examiner (MJ). Fractures were classified using the AO/OTA system [21–23]. For fractures involving the lateral pillar, body, or neck, the glenopolar angle (GPA) was measured pre- and postoperatively [24]. Fracture union was assessed using plain radiographs obtained at routine follow-up visits. Union was defined as the presence of bridging callus across the fracture site and the absence of implant loosening or secondary displacement. Computed tomography was not routinely performed and was reserved for cases with equivocal findings on plain radiographs or clinical suspicion of delayed union. Complications such as infection, wound issues, revision surgery, or implant failure were identified from clinical documentation.

### Statistical analysis

Normality was assessed using the Kolmogorov–Smirnov test. Continuous variables were compared using t-tests or Mann–Whitney U tests as appropriate. Data are presented as mean ± standard deviation. Significance was set at *P* < .05. Analyses were performed using Prism software (version 5.0.4; GraphPad) and Excel (Version 16.89.1; Microsoft).

## Results

### Patients’ and surgical characteristics

Between January 2015 and December 2019, 31 patients underwent operative treatment for a scapular fracture. Thirteen patients (42%) were lost to follow-up, one (3%) died from unrelated causes, and one (3%) was excluded due to a different posterior approach. Sixteen patients (55%) were available for analysis, with 10 (63%) undergoing outpatient follow-up and 6 (38%) being contacted via telephone or mail. Accordingly, a total of 16 patients fulfilled the inclusion criteria and were included in the final analysis. Baseline characteristics, injury mechanisms, and surgical details are summarized in Table 1. Five patients (31%) required a two-stage surgical approach due to associated injuries, including concomitant coracoid and anterior glenoid rim fractures (*n* = 3) or combined coracoid and acromion fractures (*n* = 1) or concomitant coracoid fracture alone (*n* = 1).

Two patients with associated ipsilateral clavicle fractures were treated during the same single-stage operative session (Fig. 1A).

**Table 1:** Baseline characteristics

Variable				n
Mean patient age in years [SD]				48 [14]
Mean follow-up in months [SD]				75 [16]
Gender				
Men				16 (100%)
Women				0 (0%)
Injured side				
Right				7 (44%)
Left				9 (56%)
Mean time between fracture and ORIF in days [SD]				9 [6]
Mean surgical duration in minutes [SD]				131 [50]
One-stage surgery				11 (69%)
Two-stage surgery				5 (31%)
Trauma mechanism				
Car accident				3 (19%)
Pedestrian accident				1 (6%)
Motorcycle accident				5 (31%)
Bicycle accident				4 (25%)
Fall from a height (> 2 m)				2 (13%)
Fall (< 2 m)				1 (6%)
BMI [SD]				26 [5]
ASA [SD]				1,6 [0,7]

*SD *standard deviation, *ORIF *open reduction and internal fixation*, BMI *body mass index, *ASA *amerian society of anesthesiologists classification

**Table 2:** Radiological evaluation

AO/OTA classification				*n*
B (body involvement)				15 (94%)
F0 (articular segment without Glenoid)				1 (6%)
F1 (glenoid rim fracture)				3 (19%)
F2 (multifragmentary glenoid fracture)				1 (6%)
A1 (coracoid fracture)				5 (31%)
A2 (acromion fracture)				1 (6%)
A3 (spine fracture)				1 (6%)
GPA preoperative				33° [7° SD]
GPA postoperative				34° [5° SD]
Complications				*n*
Revision surgery				1 (6%)
Infects				0
Delayed wound healing				0
Implant failure				0
Hardware removal				1 (6%)

*SD *Standard Deviation

### Functional outcomes

Functional outcomes are summarized in Table 3.

**Table 3:** Clinical Outcome Parameter

Outcome parameter				mean value ± SD
Constant score				76 ± 12
Pain				13 ± 1
ADL				13 ± 5
ROM				36 ± 5
Strength				13 ± 4
SPADI score				83 ± 16
DASH score				16 ± 15
VAS score for pain				2 ± 1
Active forward flexion				163° ± 23°
Active external rotation at 0°				70° ± 12°
Active internal rotation				L1

*SD *standard deviation, *ROM *range of motion*, SPADI s*houlder pain and disability index*, DASH *disabilities of arm, shoulder and hand*, VAS *visual analogue scale

When stratified by number of surgical stages, 11 patients underwent single-stage surgery, and five patients required staged procedures (Table 1). In the single-stage group, the mean VAS score was 1.8 ± 1.0, the mean DASH score was 15.7 ± 11.3, the mean SPADI score was 82.7 ± 14.9, and the mean CMS was 75.0 ± 10.0. In the staged surgery group, the mean VAS score was 1.2 ± 1.0, the DASH score was 17.4 ± 20.3, the SPADI score was 84.8 ± 19.4, and the CMS was 78.6 ± 15.2. Given the small and unbalanced subgroup sizes, no formal statistical comparison was performed.

### Radiographic outcomes

All fractures achieved radiographic union on plain radiographs, with a mean time to union of 12 ± 4 weeks. No delayed unions or non-unions were observed. The GPA could be determined in 14 of 16 patients (88%). Two patients were excluded due to an isolated glenoid fracture or a body fracture without lateral exit. Glenopolar angle measurements showed no significant difference between preoperative and postoperative values (Table 2).

### Complications

One patient (6%) required revision of a malpositioned coracoid screw after two-stage surgery. No infections, wound complications, or implant failures occurred during follow-up (Table 3). One patient (6%) underwent elective screw removal due to discomfort.

## Discussion

This study demonstrates favorable long-term clinical outcomes following operative treatment of scapular fractures using the tissue-sparing Brodsky approach. At a mean follow-up of more than six years, patients achieved good functional scores, low pain levels, and a largely preserved range of motion, including external rotation, even in complex fracture patterns requiring staged procedures.

These findings extend previously published results on the Brodsky approach, which have predominantly reported short- to mid-term outcomes [15]. Fandridis et al. described a mean CMS of 93.8 points after a mean follow-up of 28 months in a small cohort of six patients with extra-articular scapular fractures treated using this approach [15].

In contrast, the present study included both extra-articular and intra-articular fracture patterns and provides one of the longest follow-up periods currently available for this surgical approach.

Despite the increased fracture complexity and extended follow-up duration, the present cohort demonstrated excellent shoulder mobility, with a mean CMS range-of-motion subscore of 36 out of 40 points, and low residual pain. The lower overall mean CMS of 76 points, compared with short-term series, was primarily driven by reduced abduction strength scores rather than limitations in motion or pain. This finding suggests that long-term adaptations, aging-related changes, or fracture-related alterations in shoulder biomechanics may influence strength outcomes over time. Reduced shoulder strength following scapular fractures has been previously described [4, 18], particularly in cases involving the glenoid. It may be related to altered bony anatomy that affects the dynamic stabilizers of the shoulder, leading to earlier muscular fatigue [4, 25].

Furthermore, in six of sixteen patients (38%), strength assessment was derived from validated patient-reported outcome measures obtained via phone or mail. Although the Munich Shoulder Questionnaire has demonstrated a strong correlation with the CMS and DASH scores in shoulder pathologies [20], subjective strength ratings may underestimate true isometric strength, particularly in long-term retrospective follow-up settings, where standardized measurement conditions cannot be ensured [26–28].

Despite the increased complexity of fracture patterns included in the present study, clinical outcomes regarding range of motion and pain were favorable and comparable to those reported by Fandridis et al. This finding indicates that satisfactory functional outcomes can be achieved using a tissue-sparing posterior approach in selected scapular fractures. However, given the descriptive nature of the present study and the absence of a control group, no conclusions can be drawn regarding the superiority of this approach over alternative surgical strategies.

The mean DASH score of 16 points observed in the present cohort is consistent with values reported in previous studies assessing operatively treated scapular fractures [29]. Although patients requiring staged procedures generally presented with more complex injury patterns, descriptive outcome measures remained within a similar range to those of patients treated in a single-stage approach. However, meaningful subgroup comparisons are limited by the small sample size.

Schroder et al. reported a mean DASH score of 12 points following surgical treatment of extra-articular scapular fractures; however, the specific surgical approach was not specified [29]. Other comparative studies evaluating modified and classic Judet approaches have reported CMS values and DASH scores within a similar range, without evidence of clinically relevant differences between techniques [30].

Overall, comparisons with previously published studies are descriptive in nature and intended to provide contextual interpretation rather than comparative effectiveness. These observations must be interpreted considering the heterogeneity of fracture patterns, surgical techniques, and follow-up durations reported in the literature.

As no internal control group was included, the present study does not allow conclusions regarding the superiority of the Brodsky approach over other surgical approaches.

Both operative and non-operative treatment of scapular fractures are associated with some degree of restriction in ROM [4]. In a retrospective case series by Fandridis et al. involving six patients with extra-articular scapula fractures and a mean follow-up of 28 months, all of whom underwent ORIF for scapular fractures using the Brodsky approach, no significant loss of external rotation capability was observed compared to the uninjured shoulder [15].

The presented study corroborates these findings in a larger cohort (*n* = 16) and over a substantially longer follow-up period (mean 74.6 months). Despite increased fracture complexity, including intra-articular involvement and staged procedures, external rotation and overall shoulder mobility remained largely preserved, supporting the durability of these functional outcomes over time.

All intra-articular fractures in the present series were treated using a staged surgical strategy that included an additional anterior exposure. Staging was required due to associated coracoid fractures necessitating a separate anterior procedure and allowed direct ventral access to the intra-articular pathology. Consequently, functional outcomes observed in fractures with an intra-articular component should be interpreted in the context of the combined staged treatment strategy, rather than being attributed solely to the posterior approach.

However, it highlights that even in intra-articular scapular fractures with an associated body fracture, the body component can be addressed initially using the Brodsky approach, followed by a ventral approach for the intra-articular component if necessary. Although this two-stage procedure was preferred in the present series for concomitant coracoid fractures to ensure optimal visualization and anatomic reduction, simultaneous single-stage fixation may also be feasible in selected cases, especially for intra-articular fractures. When the intra-articular fragment can be stabilized indirectly from the dorsal aspect or when arthroscopic assistance is available, a combined approach in a single session can achieve comparable reduction quality.

Since the Brodsky approach does not require detachment of either the deltoid muscle or the infraspinatus muscle, the risk of iatrogenic soft-tissue injury is markedly reduced compared with more extensile posterior approaches. Nevertheless, potential injury to the axillary or suprascapular nerve, as well as bleeding from circumflex vessels, cannot be entirely excluded and requires careful dissection in the respective anatomical intervals.

Preservation of the posterior rotator cuff and deltoid musculature is thought to reduce the risk of postoperative restriction in shoulder range of motion, particularly external rotation. In contrast, a reduction in external rotation compared with the uninjured side has been reported following the classic Judet approach and its modifications [31, 32].

One approach to reduce the extent of the Judet approach in managing intra-articular scapular fractures may involve omitting the posterior capsulotomy. In a retrospective study, Park et al. reported on 10 intra-articular scapular fractures that underwent open reduction and internal fixation (ORIF) using the Judet approach without opening the posterior joint capsule [33]. The study reported a favorable clinical outcome with an average CMS score of 89 points and an average external rotation capability of 55.7°, with a mean follow-up of 28.6 months. Among these 10 intra-articular fractures, 3 had accompanying body fractures that were not surgically addressed. All 10 glenoid fractures were treated with screw osteosynthesis. However, only glenoid fossa fractures were included, which are known to have a better postoperative outcome compared to complex scapular fractures involving the body that also require surgical treatment [4]. Similarly, Dobelle et al. reported satisfactory mid-term results following fixation of scapular fractures via the posterior Judet approach, with a mean Constant–Murley Score of 65.8 points after a mean follow-up of 45 months, albeit with persistent limitations in shoulder strength and external rotation [32].

In comparison, the present patient cohort treated using the Brodsky approach demonstrated a slightly better CMS and superior external rotation function. However, shoulder abduction strength also remained limited in the present study. This limitation may be attributed not only to the increased complexity of the scapular fractures but also to the more extended follow-up period. Previous studies reporting good to excellent shoulder strength predominantly involved either less complex scapular fractures or shorter follow-up durations. Therefore, further studies with extended follow-up periods are needed to investigate this issue more thoroughly.

With regard to pain, a recent systematic review reported higher post-injury pain levels following non-operative treatment of scapular fractures, although some degree of postoperative pain may also persist after surgical management [4]. In the present study, average pain was evaluated using the VAS, with patients reporting a mean pain score of 2. This finding is consistent with prior reports indicating that postoperative pain following scapular fracture surgery is generally low, although some degree of pain may persist during activity [4, 18, 34, 35].

Operative treatment of scapular fractures is technically demanding but is generally associated with low complication rates when performed in experienced centers. Postoperative infections are uncommon, with reported rates ranging from 0 to 4%, and clinically relevant nerve injuries, particularly those involving the suprascapular nerve or brachial plexus, are rarely described [4, 7, 32]. In line with these reports, no infections, neurological complications, or implant failures were observed in the present cohort.

The most frequently reported postoperative issues are implant-related mechanical complaints, such as prominent plates or screws causing subacromial or thoracic irritation, which may necessitate secondary hardware removal in approximately 5–15% of cases [29, 30]. Cole et al. reported implant removal in 7 of 84 patients following operative treatment of scapular neck and body fractures, primarily due to local irritation rather than mechanical failure [7]. In the present study, one patient (6%) required revision surgery, which was related to malpositioned fixation of an associated coracoid fracture rather than failure of the scapular osteosynthesis itself, underscoring the low rate of approach- or implant-related complications.

Postoperative shoulder stiffness represents another recognized complication, particularly in the setting of delayed rehabilitation or prolonged fracture healing, with arthrolysis or manipulation under anesthesia reported in 5–15% of patients in previous series [4, 7, 30]. Notably, no cases of clinically relevant postoperative stiffness requiring intervention occurred in the present cohort, which may be attributed to stable fixation and a standardized early functional rehabilitation protocol.

Bony healing after scapular fractures is generally reliable, with reported union rates of up to 99% regardless of fracture pattern or treatment strategy, likely reflecting the excellent vascular supply of the scapular region [3, 4]. Consistent with this, all fractures in the present cohort achieved radiographic union, with no cases of delayed union or nonunion observed.

## Conclusions

At a mean follow-up of 6.3 years, operative treatment of scapular fractures using a tissue-sparing posterior approach was associated with favorable long-term functional outcomes, preserved shoulder range of motion, low pain levels, and reliable fracture union, with a low complication rate. In complex fracture patterns with associated injuries, particularly coracoid fractures, a staged surgical strategy with an additional anterior procedure may be required.

## Data Availability

The datasets generated and analysed during the current study are available from the corresponding author on reasonable request.

## Abbreviations

AO/OTA: AO Foundation / Orthopaedic Trauma Association Classification
ASA: American Society of Anesthesiologists Classification
BMI: Body Mass Index
CMS: Constant–Murley Score
CT: Computed Tomography
DASH: Disabilities of the Arm, Shoulder, and Hand
GPA: Glenopolar Angle
LCP: Locking Compression Plate
MSQ: Munich Shoulder Questionnaire
ORIF: Open Reduction and Internal Fixation
ROM: Range of Motion
SPADI: Shoulder Pain and Disability Index
SSSC: Superior Shoulder Suspensory Complex
STROBE: Strengthening the Reporting of Observational Studies in Epidemiology
VAS: Visual Analogue Scale
